# MYH9 promotes cell metastasis *via* inducing Angiogenesis and Epithelial Mesenchymal Transition in Esophageal Squamous Cell Carcinoma

**DOI:** 10.7150/ijms.46234

**Published:** 2020-07-25

**Authors:** Bin Yang, Huijuan Liu, Yanghui Bi, Caixia Cheng, Guodong Li, Pengzhou Kong, Ling Zhang, Ruyi Shi, Yunkui Zhang, Rongsheng Zhang, Xiaolong Cheng

**Affiliations:** 1 The Department of Thoracic Surgery (Ⅲ), Shanxi Cancer Hospital, Taiyuan, Shanxi 030013, P.R. China.; 2 Department of Pathology & Shanxi Key Laboratory of Carcinogenesis and Translational Research of Esophageal Cancer, Shanxi Medical University, Taiyuan, Shanxi 030001, P.R. China.; 3 Department of Pathology, The First Hospital, Shanxi Medical University, Taiyuan, Shanxi 030001, P.R. China.; 4 Department of Otorhinolaryngology, Shanxi Provincial People's Hospital Affiliated to Shanxi Medical University, Taiyuan, Shanxi 030012, P.R. China.; 5 Department of Otolaryngology-Head and Neck Surgery, Shandong Provincial ENT Hospital Affiliated to Shandong University, Jinan, Shandong 250000, P.R. China.

**Keywords:** MYH9, ESCC, angiogenesis, EMT

## Abstract

Non-muscle myosin heavy chain 9 (*MYH9*) is one novel low frequency mutated gene identified in esophageal squamous cell carcinoma (ESCC) using next-generation sequencing. However, its clinical relevance, potential function and mechanisms remain elusive.

**Methods:** Genomic sequencing datas from 104 esophageal squamous cell carcinoma (ESCC) cases were screened a series of low frequency mutant genes. *MYH9* was selected to further analyze its clinical significance, function and PCR-array was performed to explore its potential mechanism.

**Results:** *MHY9* is a low frequency mutant gene with a mutation frequency of 2.88% in ESCC. Immunohistochemical analysis showed that MYH9 expression was significantly higher in ESCC tumor tissues, and the expression levels were associated with lymph node metastasis of ESCC patients. Moreover, we found that *MYH9* knock-down led to inhibition of cell migration and invasion. PCR-array showed *MYH9* knockdown led to a significant change of genes expression associated with angiogenesis and epithelial-to-mesenchymal transition (EMT). This observation is further confirmed in TCGA database of LUSC (lung squamous cell carcinoma), CESC (cervical squamous cell carcinomas) and HNSC (head and neck squamous cell carcinoma).

**Conclusions:** Collectively, our study identifies a novel role and mechanism of *MYH9*, highlights a significance of *MYH9* as a metastatic biomarker, and offers potential therapeutic targets for ESCC patients harboring *MYH9* mutations.

## Introduction

Esophageal squamous cell carcinoma (ESCC) is the most common histological type of esophageal cancer, which ranks the sixth worldwide in terms of lethality. In China, there are about 478,000 new cases of ESCC and 75,000 deaths every year 1. In regard to the area, the Taihang Mountain region which is the junction of Shanxi, Henan and Hebei provinces, as well as the Fujian-Guangdong region, are the top 2 areas of ESCC morbidity 2. Due to the lack of specific indicators for early clinical diagnosis and effective treatment methods, its 5-year survival rate is only between 15-25% 3, while only 10% of the patients at stage III can step over the 3-year survival period, and the rate falls again to 5% in the case of the patients at stage IV 4.

Over the past decades, researchers have found mutations in several genes, such as *TP53* 5, *NOTCH1* 5, and *PIK3CA* 6, show a relatively close relationship with the appearance and development of ESCC by using next-generation sequencing technology. However, these identified gene mutations cannot provide a clear explanation on the pathogenesis mechanism.

Next-generation sequencing technology is able to accurately detect the whole genomic information of cancer cells in great detail, and is especially advantageous for the discovery of new, low frequency mutant genes by bioinformatics analysis of obtained data. In fact, significant mutant genes screened as the "driver genes" in tumorigenesis with the help of bioinformatics and statistical analysis owing to their mutation frequency is not lower than a certain threshold value 7. While a large number of mutant genes are regarded as "passenger genes" due to their low frequency. As a consequence, little research in this regard has been done, leaving their function even unknown. *MYH9* as a low frequency mutated gene was found in our study using genomic sequencing data of 104 pairs of ESCC tumor and normal samples from China. It was reported that may contribute to the progression and poor prognosis of ESCC, and this effect may be associated with increased cancer cell migration 8, 9. However, the detailed function and mechanism of *MYH9* in ESCC are still unknown.

In this study, our results showed that *MYH9* was significantly increased in ESCC tissues compared to paired normal tissues. And decreased *MYH9* expression was associated with lymph node metastasis of ESCC patients. Additionally, we found loss-function of *MYH9* leads to inhibition of ESCC cell migration and invasion. Importantly, we performed PCR-array in *MYH9* knockdown ESCC cells and matched NC cells and together with the available TCGA database, we validated the associations among *MYH9* and the significant changed genes of angiogenesis and epithelial-to-mesenchymal transition (EMT) pathways in ESCC and other squamous carcinomas. Our study identifies a novel role and mechanism of *MYH9* contributes to ESCC progression, provide several possible therapeutic targets for ESCC patients harboring *MYH9* mutations.

## Materials and Methods

### Samples and clinical information

In this research, tumor and adjacent normal tissue samples of patients were obtained from 104 ESCC patients recruited from the ethics committee of Shanxi Cancer Hospital and Henan Cancer Hospital. 90 samples WES and 14 samples WGS were performed on all of the tumor tissues from these 104 patients as well as on matched paracancer tissue. Sequencing data and clinical characteristics of the analyzed samples were presented in our previously published study 10 and available for download from the European Genome-phenome Archive (EGA) under accession number EGAS00001001487. The human tissue array (Cat No.: HEso-Squ172Sur-02) for MYH9 protein detection was bought from Shanghai Outdo Biotech Co.,Ltd. MYH9 mutation information in various of tumors was obtained from ICGC database (https://www.ncbi.nlm.nih.gov/pmc/articles/PMC3263593/) and COSMIC database (https://www.ncbi.nlm.nih.gov/pmc/articles/PMC2705836/ ).

### Cell lines

All of the esophageal cancer cell lines, including KYSE140, KYSE180, ECA109, KYSE410, KYSE510, KYSE150, and TE1 were stored at the Translational Medicine Research Center of the Shanxi Medical University (Taiyuan, China). All of the cells were incubated in the RPMI-1640 medium containing 10% fetal bovine serum (FBS) at 37 °C with 5% carbon dioxide.

### MYH9 knockdown

ESSC cell lines KYSE140 and KYSE180 with high endogenous expression of MYH9 were selected for the MYH9 knockdown. Specifically, two independent siRNAs were cloned into the PLKO.1-puro carriers. In order to package the lentivirus, HEK293T cells were transfected using the lentiviral vector and packaging carrier, including pMD2.G and psPAX2 via Lipofectamine 2000. After 48 h of transfection, the viral supernatant was collected and filtered with 0.22 μm filters to prepare medium with appropriate concentration. The infected cells were then incubated at 37 °C. After 24 h, the fusion degree was around 50-60%. Fresh medium containing the virus was then added. After 48 h of infection, 4 mg/ml puromycin was applied to select cells. Finally, qPCR was utilized to analyze the interference efficiency of *MYH9*.

### MTT experiments

Cultured cells were harvested, resuspended, and seeded on 96-well plates with a cell concentration of 5×10^3^/ cells. These cells were cultured for 24 h, 48 h, 72 h, and 96 h under normal conditions. Furthermore, 20 μl of 5 mg/ml MTT were added into the medium. After 4 h of incubation at 37 °C, the suspension was removed. A total of 150 μl of DMSO were added into every well in order to dissolve the generated crystals. The microplate reader was utilized to examine cell absorbance values at 490 nm. Every group contained five samples and every experiment was repeated at least three times.

### Invasion and migration experiments

The transwell assay was used to explore cellular invasion and migration. Specifically, 5×10^4^ cells were seeded in the upper chamber in basic medium without FBS, with an addition of 600 μl of RPMI-1640 medium with 10% FBS to the lower chamber. After 48 h of incubation, 4% paraformaldehyde was added to the upper chamber to fix the cells. A 0.1% solution of crystal violet was used for staining. When samples were observed under a microscope, five random areas were selected for counting cell numbers. The invasion experiments were performed in a similar fashion, with an addition of 50 μl of the matrix gel in every well before cell seeing.

### qPCR

The level of gene expression in ESCC cell lines was examined by qPCR. The total RNA was extracted by RNAiso. After the introduction of the SYBR Green Premix Ex TaqTM, qPCR experiments were performed using the Applied Biosystems StepOnePlus system. GAPDH served as a reference gene in order to calculate the relative expression of target genes. All qPCR experiments contained a negative control group without any template. Every test was repeated at least three times.

### Immunohistochemical analysis

The detection of MYH9 protein in tissue array was achieved by IHC. The paraffin sections were incubated overnight with an appropriate antibody at 4 °C. After the PV8000 and DAPI staining were complete, hematoxylin was utilized to counterstain. Images were captured at 1003. The amount of the protein of interest was analyzed with Aperio Cytoplasma 2.0 software.

### Vascular mimicry

A total of 50 μl of the matrigel were added into 96-well plates and kept at 37 °C for 30 min to ensure gel formation. Then ESCC cell lines (5× 10^5^) in 50 μl of complete medium (consist of 200 μl FBS, 4 μl Hydrocortisone, 40 μl hFGF-B, 10 μl VEGF, 10 μl R3-IGF-1, 10 μl Ascorbic Acid, 10 μl hEGF, 10 μl GA-1000, 10 μl Heparin in 10 ml EGM medium) were added to each well and incubated at 37 °C, 5% CO_2_ for 12 h. Ten random fields of view were selected to count the number of tube-like structures.

### Statistical analysis

According to the receiver operating characteristic curve (ROC) analysis, the optimal cut-off value of 1 of MYH9 protein level (T-N) was selected with higher sensitivity and specificity to divide all ESCC cases into two groups: MYH9 low and MYH9 high. Rank sum and Chi square (χ^2^) tests were utilized to analyze correlation between the MYH9 expression level and clinical ESCC data. Kaplan-Meier estimation and log-rank test were used to perform the subsistence analysis in groups with different expression levels of MYH9. Differences between groups were determined by Student's t-test with subsequent Bonferroni correction, with *P*<0.05 considered significant. SPSS 19.0 was used to perform statistical analysis.

## Results

### Non-muscle myosin heavy chain 9 (*MYH9*) is a low frequency mutant gene detected by next-generation sequencing

**Figure 1:**
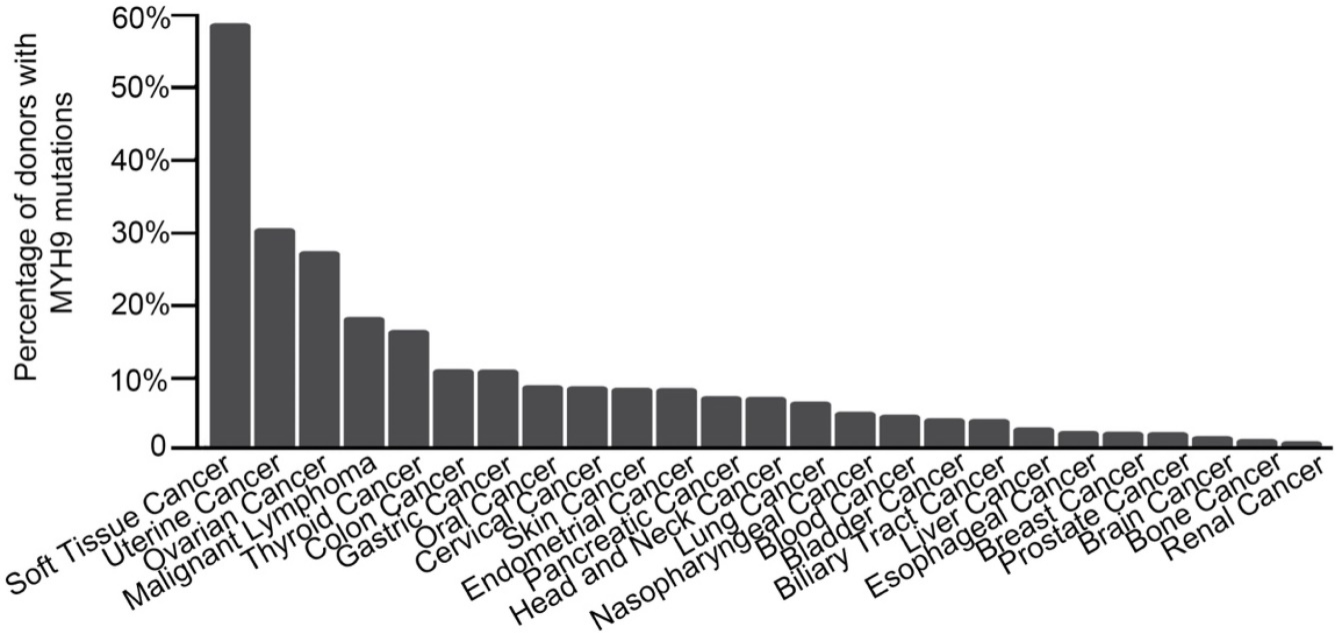
***MYH9* mutation frequency in various cancers.** Data obtained from the ICGC database. The X-axis represents cancer types; the Y-axis shows *MYH9* mutation frequency.

In our previous research, the whole genome and exome sequencing was implemented on 14 ESCC cases from Shanxi and 90 ESCC cases from Henan. A total of 8 significant mutant genes (FDR<0.178, *P*<0.0001) and a series of low frequency mutant genes were identified 10. Among them, *MHY9* is a low frequency mutant gene that exhibited 6 mutations in 3 cases, with a mutation frequency of 2.88% (3/104). The analysis result of ICGC database showed that *MYH9* was mutated in multiple common tumors (**Figure 1**). Moreover, we found that 90% (223/248) mutations of *MYH9* were located in the CDS region in COSMIC database. Therefore, we hypothesized that the *MYH9* mutation is closely related to carcinogenesis.

### Elevated MYH9 expression is associated with lymph node metastasis

**Figure 2:**
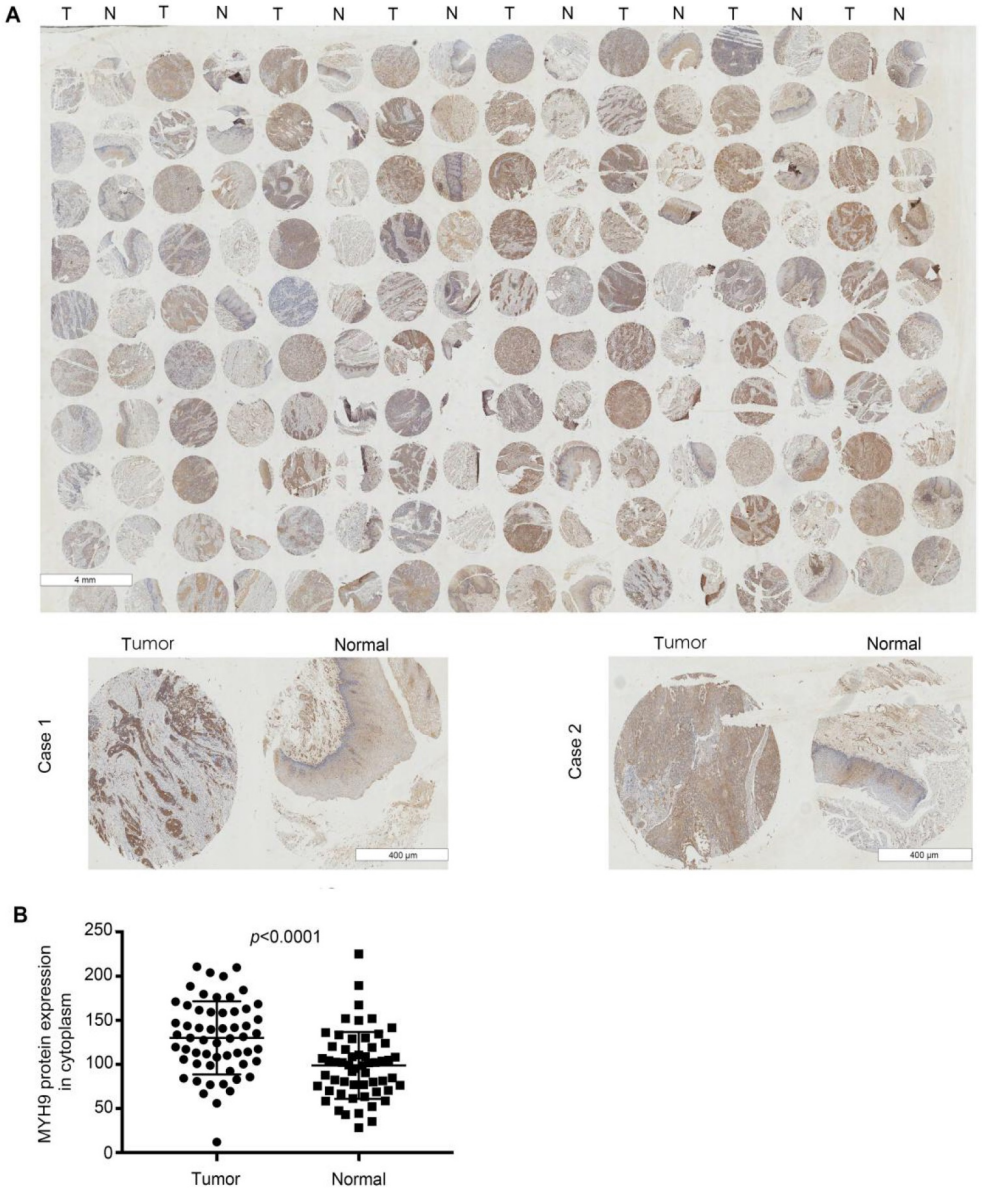
** MYH9 was frequently up-regulated in ESCC tissues compared to that of adjacent normal tissues. (A)** Representative immunohistochemistry images of MYH9 expression in tumor tissues and adjacent normal tissues from paraffin-embedded formalin- fixed ESCC tissue microarrays containing 57 tumors and corresponding non-tumor tissues. Scale bars represent 4 mm (upper) and 400 µm (lower). **(B)** Comparison of MYH9 protein level in paired ESCC tumor tissues and normal tissues based on TMA data (n = 57, Rank sum test, p < 0.001).

Next, we analyzed the expression level of MYH9 by immunohistochemical analysis based on tissue microarray including another 57 of ESCC tumor tissues and paired normal tissues. Our results demonstrated that MYH9 protein showed strong cytoplasm staining in esophageal carcinoma tissues whereas nearly negative in matched normal tissues (**Figure 2A**). A significant statistical difference was found between the two groups (*p*<0.001) (**Figure 2B**). Moreover, the MYH9 expression level is related to lymph node metastasis. Age, histological grading, clinical stage, and history of alcohol use were considered to be not significantly related to MYH9 expression level (**Table 1**).

**Figure 3:**
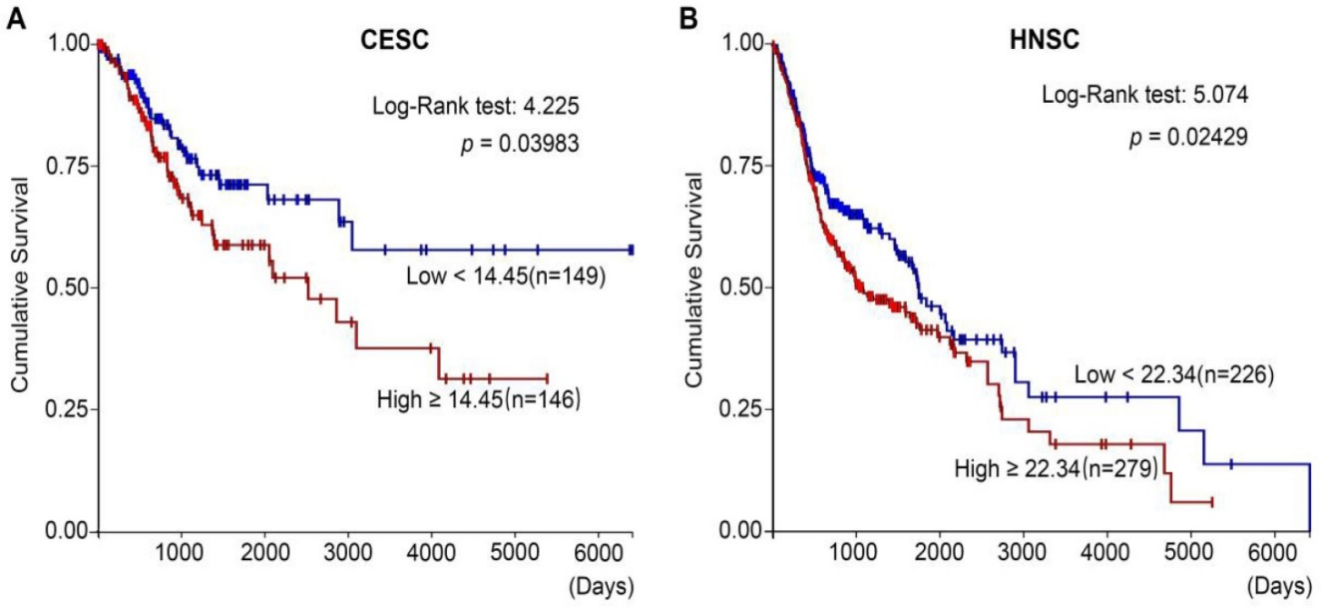
** MYH9 correlates with poor prognosis in CESC and HNSC. (A)** Kaplan-Meier survival curves of CESC patients with different MYH9 level in overall population. **(B)** Kaplan-Meier survival curves of HNSC patients with different MYH9 level in overall population.

In addition, after analyzing the TCGA (The Cancer Genome Atlas) databases of CESC (cervical squamous carcinoma) (**Figure 3A**) and HNSC (Head and neck squamous cancer) (**Figure 3B**), we found that high MYH9 expression level was strongly associated with shortened survival period of patients. Therefore, high MYH9 expression may be one of the most important markers in prognosis of squamous cell carcinoma.

### *MYH9* knockdown can inhibit migration and invasion capability of esophageal cancer cells

*MYH9* encodes non-muscle myosin II A (NMIIA) containing an IQ domain and a myosin globular head domain, both of which are associated with some important cellular functions such as cytoplasmic division, cell migration, and maintenance of cell shape. *MYH9* has been found to play an important role in hereditary diseases, thrombosis, hearing impairment, inflammation, and tumor metastasis 11, 12. It is also a candidate oncogene for breast cancer and plays an important role in metastasis 13. However, the function and mechanisms of *MYH9* in ESCC have not been widely investigated in the literature.

**Figure 4:**
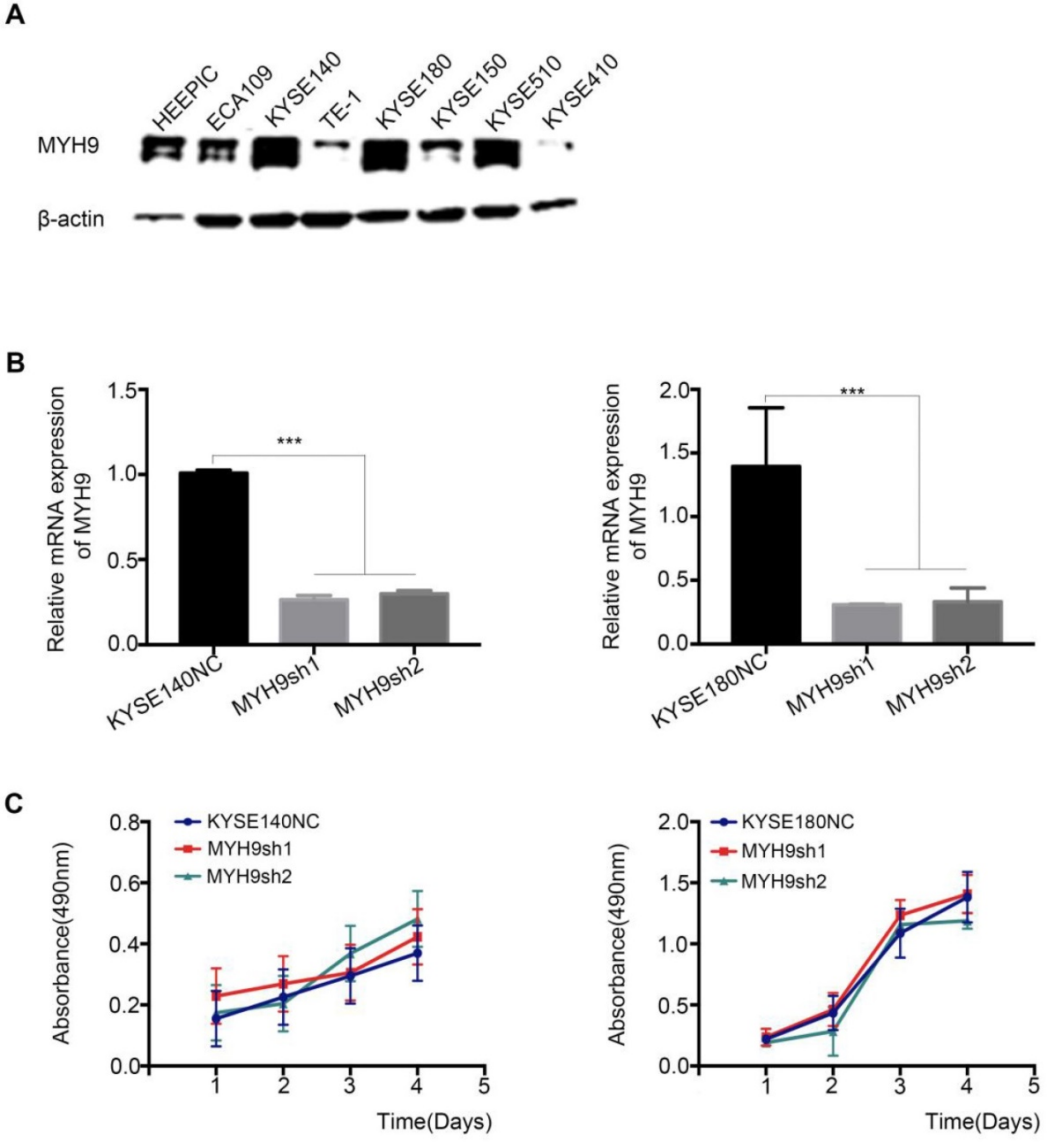
***MYH9* has not affect on ESCC cell proliferation**. **(A)** The protein expression pattern of MYH9 in eight of ESCC cell lines detected by western blot. **(B)** Knockdown efficiency of *MYH9* in KYSE140 and KYSE180 cells were tested by qPCR. **(C)** No changes of KYSE140 and KYSE180 cells proliferation upon knockdown of *MYH9*. All data are presented as the mean ± standard deviation and three independent experiments. ***p < 0.001.

**Figure 5:**
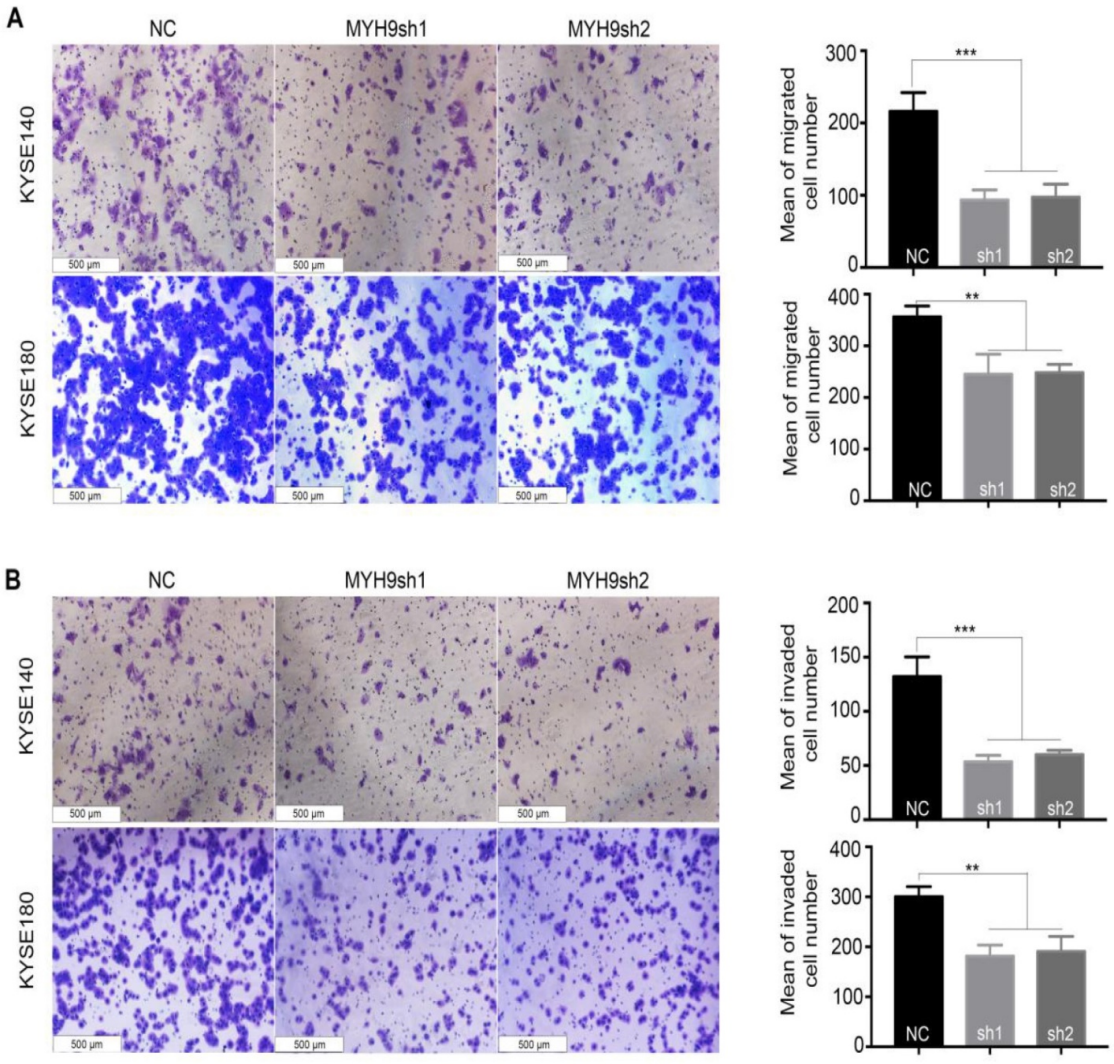
***MYH9* acts as an oncogene affecting ESCC cell migration and invasion.** *MYH9* knockdown markedly inhibited KYSE140 and KYSE180 cell migration **(A)** and invasion** (B)**. All data are presented as the mean ± standard deviation and three independent experiments. *p < 0.05, **p < 0.01, ***p < 0.001.

An RNAi experiment was performed in the endogenous esophageal squamous carcinoma cell line (KYSE140 and KYSE180) with high expression of MYH9 (**Figure 4A and 4B**). The result showed that the decreased expression of MYH9 had no effect on the proliferation of KYSE140 and KYSE180 (**Figure 4C**), but the cell migration and invasion activity were significantly inhibited (**Figure 5**), suggesting that *MYH9* may be a critical oncogene of tumor metastasis of ESCC.

### Screening genes and pathways related to *MYH9* gene expression in ESCC cells

**Figure 6:**
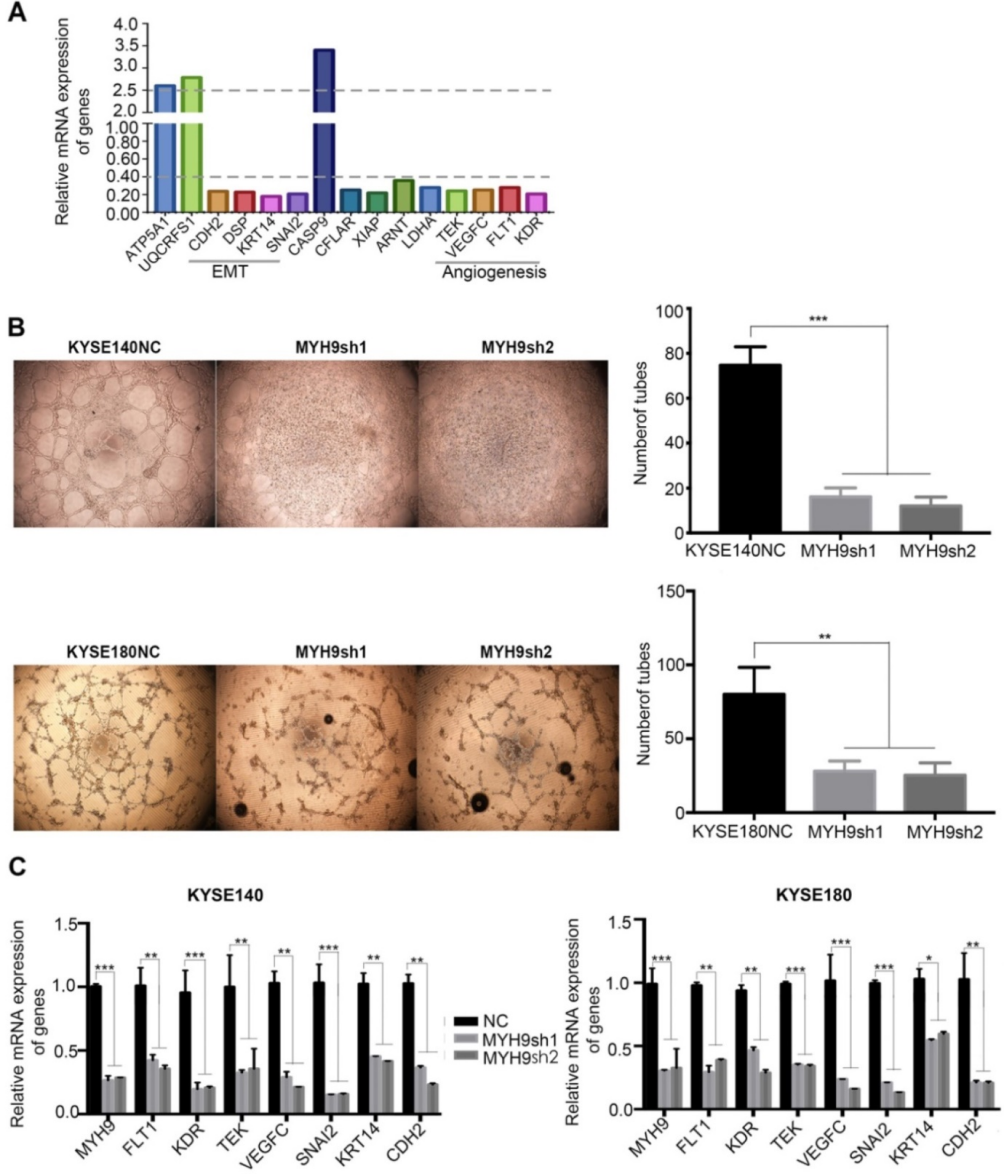
** Key cancer pathway components altered in *MYH9* knockdown cells. (A)**PCR-Array was used to show the key cancer pathway correlating with *MYH9*. **(B)** Left panel: representative images of *in vitro* vasculogenic mimicry tube formation assay. Right panel: the quantification results of *in vitro* vasculogenic mimicry tube formation using Image J software. **(C)**q-PCR was used to detect the mRNA level of *FLT1*, *KDR*, *TEK*, *VEGFRC*, *SNAI2*, *KRT14* and *CDH2* in KYSE140NC (left), KYSE180NC (right) and *MYH9*sh. *GAPDH* was used as a loading control. All data are presented as the mean ± standard deviation and three independent experiments. *p < 0.05, **p < 0.01, ***p < 0.001.

To explore the specific mechanism *MYH9* utilizes as an oncogene in ESCC, PCR-array experiments were performed for a comparative analysis between the KYSE140 and KYSE140-*MYH9*sh cells. A total of 15 differentially expressed genes were identified (fold change ≥2.5), including 3 up-regulated genes (*ATP5A1*, *CASP9* and *UQCRFS1*) and 12 down-regulated genes (*FLT1, KDR, DSP, CFLAR, KRT14, LDHA, SNAI2, TEK, VEGFC, XIAP, ARNT* and *CDH2*). These genes were mainly distributed in the angiogenesis and epithelial-mesenchymal transition (EMT) related signaling pathways, suggesting that the *MYH9* functional mechanism is related to these pathways (**Figure 6A**).

### *MYH9* can inhibit angiogenesis and molecular phenotype of EMT in ESCC

Consistent with the result of PCR-Array, we used vasculogenic mimicry *in vitro* to determine whether *MYH9* mediate the morphological alteration of the ESCC cells. Our results demonstrated that angiogenesis was inhibited after *MYH9* knockdown in ESCC cells, directly indicating the promotion effect of *MYH9* on angiogenesis (**Figure 6B**). And, we detected with the help of qPCR the changes in expression of angiogenesis and EMT-related markers in knockdown of *MYH9* and its control cells of ESCC. The results showed that after knocking down *MYH9*, the expression of angiogenesis markers *FLT1*, *KDR*, *TEK*, and *VEGFC* decreased, and the expression of mesothelial cell markers *SNAI2*, *KRT14* and *CDH2* decreased (**Figure 6C**).

**Figure 7:**
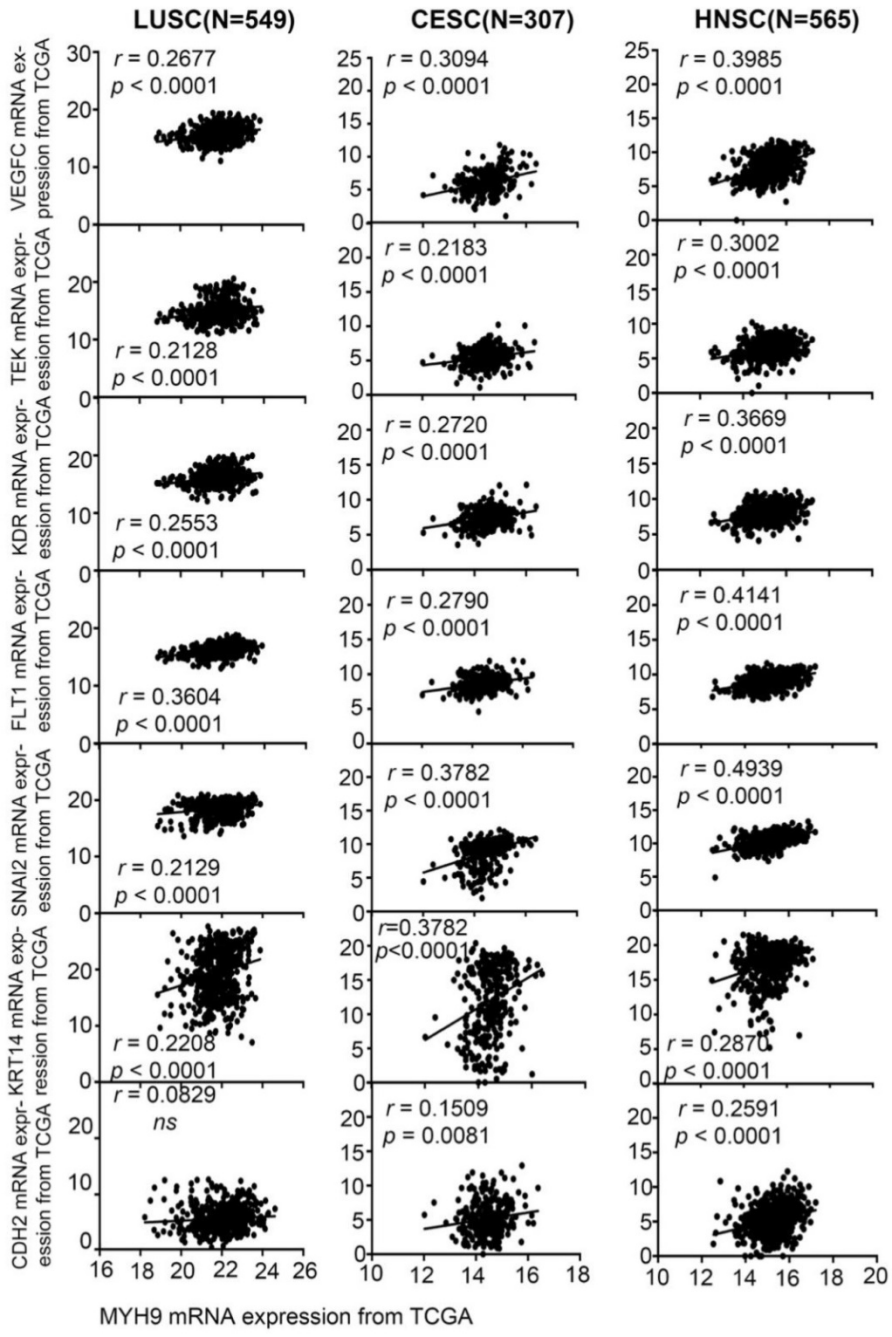
***MYH9* was positive correlated with *FLT1*, *KDR*, *TEK*, *VEGFC*, *SNAI2*, *KRT14* and *CDH2* in squamous cell carcinoma.** *MYH9* was positive correlated with *FLT1*, *KDR*,* TEK*, *VEGFC*, *SNAI2*, *KRT14* and *CDH2* in 549 LUSC samples based on TCGA data (1^st^ column), in 307 CESC samples based on TCGA data (2^nd^ column), in 565 HNSC samples based on TCGA data (3^rd^ column).

We analyzed the correlation between *MYH9* and the expression of angiogenesis and EMT markers in 549 LUSC (lung squamous cell carcinoma) (**Figure 7,1^st^ column**), 307 CESC (**Figure 7,2^nd^ column)**, and 565 HNSC (**Figure 7,3^rd^ column**) in the TCGA database. It was found that *MYH9* was significantly positively correlated with angiogenesis markers *FLT1*, *KDR*, *TEK*, and *VEGFC*, significantly positively correlated with EMT markers *SNAI2*, *KRT14* and *CDH2*. The above-mentioned data suggest a possible mechanism of action of *MYH9*. Combined with our results, it is suggested that* MYH9*, as a low-frequency mutant gene in ESCC, can regulate the metastasis of ESCC through angiogenesis and EMT pathway.

## Discussion

In previous research, whole genome and exome sequencing were used to analyze 104 cases of ESCC and matched normal tissues. A series of ESCC-related mutant genes, including some star genes (*NOTCH1, TP53,* and *PIK3C*) and some low frequency mutant genes were identified 10, 14. *MYH9* was one of the low frequency mutant genes discovered during the sequencing, with 6 mutations in 3 cases, and the mutation frequency is about 2.88% (3/104). Moreover, the mutant *MYH9* was found in a range of common tumors, and 90% (223/248) of the *MYH9* mutations were located in the CDS region. Therefore, it is reasonable to believe that a mutation of *MYH9* plays an important role in the development of cancer.

The MYH9 gene encodes the non-muscle myosin IIA heavy chain, which is located on chromosome 22q11.2 and consists of 1960 amino acids with a molecular weight of 226 KD 15. This protein is widely expressed in a variety of tissues and cells. Myosin 9, one of the most important cytoskeleton constituents, takes part in a number of cellular functions, including cell division, cell migration, and cell shape maintenance. According to our research results, MYH9 exhibited a relatively high expression in ESCC tissues, showing significant correlation with lymph node metastasis. Moreover, down-regulating the MYH9 expression inhibited angiogenesis, migration, and invasion of esophageal cancer cells. Consistent with our research results, it was reported that expression of MYH9 is closely related to the malignant degree of cancer, and can be regarded as a marker for evaluating lymph node metastasis and poor prognosis in breast cancer 16, epithelial ovarian cancer 17 and acute myeloid leukemia 18. Combined detection of vasculogenic mimicry, MYH9 and E-cad may play an essential role in predicting the invasion, metastasis, and progression of patients with ESCC 8, 9. Moreover, Wang et al. reported that *MYH9* promoted tumorigenesis by regulating MAPK/AKT signaling in colorectal cancer 19. It also promoted tumor growth and metastasis by activating the Wnt/β-catenin signaling pathway and EMT in pancreatic cancer 20. By interfering with the expression of *MYH9*, the morphology, adhesion, and cytoskeleton of both HeLa and HEK293 cells could be modified 21.

According to the PCR-array results, *MYH9* was shown to take part in the process of ESCC migration through angiogenesis and EMT signaling pathways, while both angiogenesis and EMT are important factors in tumor development. *MYH9* knockdown cells exhibited angiogenesis-related gene changes, including *FLT1, KDR, TEK,* and *VEGFC.* It can be well known that the 3 main vascular endothelial growth factor (VEGF) receptors are VEGFR1, VEGFR2, and VEGFR3. Based on previous reports, the main capability of VEGFR1 encoded by *FLT1* is regulating the rearrangement of cytoskeleton, which can induce cell migration. FLT1 can combine with VEGFR-A, VEGFR-B, and FGF-2, promoting proliferation of endothelial cells in the process of angiogenesis in various cancer 22-24. As an important receptor for VEGF signal transduction, the vascular endothelial growth factor receptor-2 (VEGFR-2) encoded by *KDR*, highly expressed in most tumors, can not only promote the proliferation of vascular endothelial cells, induce angiogenesis around tumor tissue, but also accelerate the migration of cancer cells 25, 26. TEK as a cell surface receptor for ANGPT1, ANGPT2, and ANGPT4, plays an important role in regulating angiogenesis, migration, and adhesion of endothelial cells. The combination of ANGPT1 and TEK can enhance the connections among endothelial cells and promote the mutation and stability of newborn blood vessels 27. In contrast, ANGPT2 can inhibit TEK activity, which damages blood vessel stability and promotes the development of VEGF-dependent blood vessel development 28. At the same time, the changes in the expression of EMT-related markers, including the known *CHD2,* as well as *SNAI2* and *KRT14* were also observed. The induction of EMT by SNAI2 can not only promote the invasion ability in cancer cells, but also lead to drug resistance, pressure, and immune response 29. In liver cancer, SNAI2 can control multidrug resistance by inhibiting the expression of ABC transporter gene 30. Moreover, several previous studies demonstrated that expression of CDH2 has a positive correlation with SNAI2 in tumor tissues. Encoded by the *CDH2* genes, N-CAD is expressed in ganglial cells, myocardium and mesothelial cells, and associated with an increased risk of invasive cancer. The aberrant expression of N-cadherin was significantly related to the differentiated degree, histological type, invasion and metastasis of gastric cancer 31. KRT14 as a basal epithelial marker promoted the invasive capacity, but had no impact on cell viability or proliferation, suggesting an invasion-specific role 32. In addition, according to the correlation analysis of *MYH9* expression and related genes using the TCGA databases of squamous cell carcinoma, a positive correlation was found between the expression of *MYH9* and *FLT1, KDR, TEK, VEGFC, SNAI2 ,KRT14* and *CHD2* in LUSC, CESC and HNSC. These results indicated that *MYH9* could regulate the migration of ESCC via angiogenesis and EMT.

In conclusion, a low frequency mutant gene related to ESCC was found to play an important role in metastasis and angiogenesis of esophageal cancer cells. Correlation between MYH9 and lymph node metastasis indicated that *MYH9* can be regarded as a potential therapy target, providing a foundation for clinical diagnosis and molecular therapy in ESCC patients.

## Figures and Tables

**Table 1:** Correlation between the expression level of myosin IIA and clinicopathological factors in esophageal squamous cancer

Variable	Total (n=57)	MYH9 expression		*P*
		Low (n=12)	High (n=45)	
**Gender**				
Female	16	4	12	0.723
Male	41	8	33	
**Age**				
≤60 years	23	3	20	0.325
>60 years	34	9	25	
**Depth of invasion**				
T1/2	21	5	16	0.744
T3/4	36	7	29	
**Lymph node metastasis**				
Negative	31	10	21	0.047*
Positive	26	2	24	
**Histological grade**				
G1	11	4	7	0.373
G2	39	7	32	
G3	7	1	6	
**TNM staging**				
I/II	36	10	26	0.177
III	21	2	19	

*P < 0.05; P-values analyzed by Fisher's exact test or χ^2^ test.
